# Supplementary material for: Comprehensive evaluation of EEG spatial sampling, head modelling and parcellation effects on network alterations in idiopathic generalized epilepsy
Source: Brain Commun. 2026 Jan 27;8(1):fcag022. doi: 10.1093/braincomms/fcag022 (PMC12917543; doi:10.1093/braincomms/fcag022)
Supplement: fcag022_Supplementary_Data [file fcag022_supplementary_data.docx]

Supporting Information to

**Comprehensive evaluation of EEG spatial sampling, head modeling, and parcellation effects on network alterations in idiopathic generalized epilepsy**

Christina Stier^1, 2, 3^, Markus Loose^1^, Carmen Loew^1^, Marysol Segovia Oropeza^1^,

Sangyeob Baek^4^, Holger Lerche^2^, Niels K. N. Focke^1,2^

*^1^ Clinic of Neurology, University Medical Center Göttingen, Göttingen, Germany*

*^2^ Department of Neurology and Epileptology, Hertie Institute for Clinical Brain Research, University of Tübingen, Tübingen, Germany*

*^3^ Institute for Biomagnetism and Biosignalanalysis, University of Münster, Münster, Germany*

*^4^ MEG-Center, Hertie Institute for Clinical Brain Research, University of Tübingen, Tübingen, Germany*


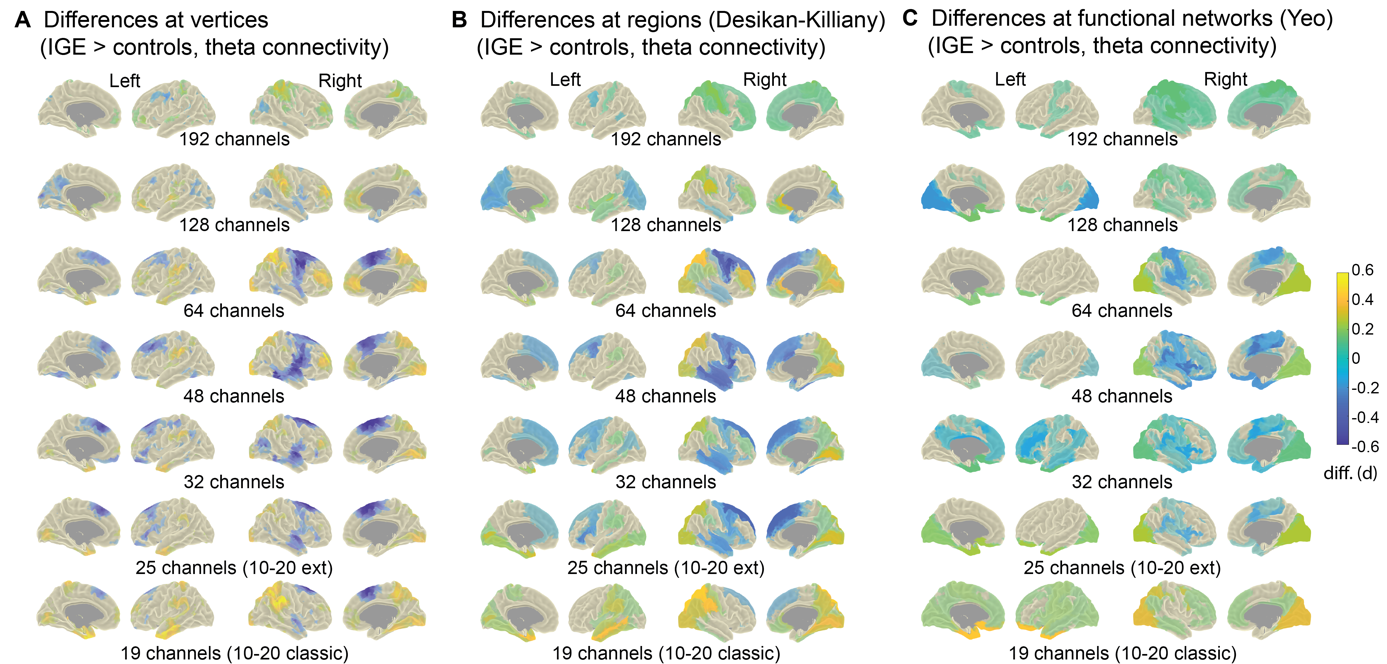


**Supplementary Figure 1** Deviations from the original effect size maps (256 channels, individual head models) for connectivity at different channel sets and parcellations

Statistical group comparisons (IGE > controls) were performed separately for different parcellations of the brain (vertices, anatomical regions, and functional networks) based on permutation analysis of linear models, including age, sex, and scanner site as covariates (n_IGE_ = 35; n_controls_ = 54). Standardized effect sizes (Cohen d) were calculated based on the resulting t-values corrected for the influence of the covariates. For each resolution, the differences between the Cohen d-maps for the 256 channel-layout and those for the reduced layouts (19 to 192 channel densities) were then calculated and color-coded (**A-C**). Green and yellow colors indicate larger effect sizes and blue colors depict smaller effect sizes in the 256-reference map than in the respective lower sampling map. The results shown in **(A)** rely on EEG signals projected to 2004 vertices using individual head models and different channel compositions. For the anatomical **(B)** and network-based **(C)** analyses, individual connectivity values at the vertices were averaged for each region of interest (Desikan et al., 2006; Yeo et al., 2011). diff.(d) = differences in Cohen’s d value.


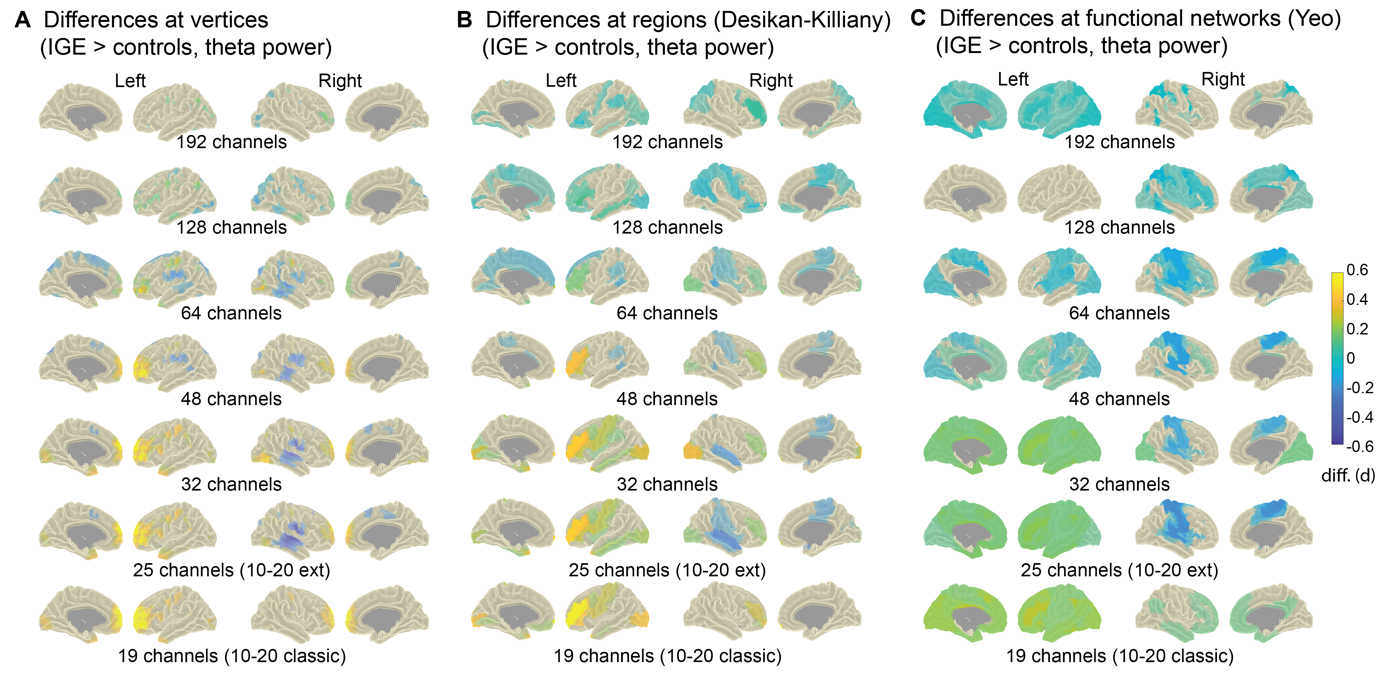


**Supplementary Figure 2** Deviations from the original effect size maps (256 channels, individual head models) for power at different channel sets and parcellations

Statistical group comparisons (IGE > controls) were performed separately for different parcellations of the brain (vertices, anatomical regions, and functional networks) based on permutation analysis of linear models, including age, sex, and scanner site as covariates (n_IGE_ = 35; n_controls_ = 54). Standardized effect sizes (Cohen d) were calculated based on the resulting t-values corrected for the influence of the covariates. For each resolution, the differences between the Cohen d-maps for the 256 channel-layout and those for the reduced layouts (19 to 192 channel densities) were then calculated and color-coded (**A-C**). Green and yellow colors indicate larger effect sizes and blue colors depict smaller effect sizes in the 256-reference map than in the respective lower sampling map. The results shown in **(A)** rely on EEG signals projected to 2004 vertices using individual head models and different channel compositions. For the anatomical **(B)** and network-based **(C)** analyses, individual power values at the vertices were averaged for each region of interest (Desikan et al., 2006; Yeo et al., 2011). diff.(d) = differences in Cohen’s d value.

**Supplementary References**

Cohen, J. (1992). A power primer. *Psychological bulletin*, *112*(1), 155.

Desikan, R. S., Ségonne, F., Fischl, B., Quinn, B. T., Dickerson, B. C., Blacker, D., Buckner, R. L., Dale, A. M., Maguire, R. P., & Hyman, B. T. (2006). An automated labeling system for subdividing the human cerebral cortex on MRI scans into gyral based regions of interest. *Neuroimage*, *31*(3), 968-980.

Oostenveld, R., Fries, P., Maris, E., & Schoffelen, J.-M. (2011). FieldTrip: open source software for advanced analysis of MEG, EEG, and invasive electrophysiological data. *Computational intelligence and neuroscience*, *2011*.

Yeo, B. T., Krienen, F. M., Sepulcre, J., Sabuncu, M. R., Lashkari, D., Hollinshead, M., Roffman, J. L., Smoller, J. W., Zöllei, L., & Polimeni, J. R. (2011). The organization of the human cerebral cortex estimated by intrinsic functional connectivity. *Journal of neurophysiology*.
